# Evaluating Neutralizing Antibodies in Hantavirus-Infected Patients Using Authentic Virus and Recombinant Vesicular Stomatitis Virus Systems

**DOI:** 10.3390/v17050723

**Published:** 2025-05-19

**Authors:** Punya Shrivastava-Ranjan, Jamie A. Kelly, Laura K. McMullan, Deborah Cannon, Laura Morgan, Payel Chatterjee, Shilpi Jain, Joel M. Montgomery, Mike Flint, César G. Albariño, Christina F. Spiropoulou

**Affiliations:** Viral Special Pathogens Branch, Division of High Consequence Pathogens and Pathology, Centers for Disease Control and Prevention, Atlanta, GA 30329, USA; twz3@cdc.gov (J.A.K.); lim8@cdc.gov (L.K.M.); mwe9@cdc.gov (P.C.);

**Keywords:** hantaviridae, orthohantavirus, Sin Nombre virus, Andes virus, hemorrhagic fever

## Abstract

Hantaviruses, including the Sin Nombre virus (SNV) and Andes virus (ANDV), are associated with severe global health risks, causing high mortality rates in hantavirus pulmonary syndrome (HPS) patients. Neutralizing antibodies are essential for virus clearance and survival, making neutralization assays critical for understanding immunity and evaluating therapeutic strategies. In this study, we developed a recombinant vesicular stomatitis virus (VSV)-based surrogate system expressing SNV and ANDV glycoproteins (GPCs), enabling neutralization studies under biosafety level 2 conditions. The neutralization titers obtained with the VSV-based system closely matched the findings from authentic hantavirus assays performed under biosafety level 3 conditions, confirming its potential as a useful tool for determining immune responses and advancing hantavirus research.

## 1. Introduction

Hantaviruses, members of the family *Hantaviridae*, are responsible for severe zoonotic diseases, including hantavirus pulmonary syndrome (HPS) and hemorrhagic fever with renal syndrome. The Sin Nombre virus (SNV) and Andes virus (ANDV) are among the most significant pathogens, with the SNV being the primary cause of HPS cases in North America [1,2,3,4]. In South America, the ANDV is notable for its unique capability for person-to-person transmission. Immunological responses, particularly neutralizing antibody responses, are critical for controlling hantavirus infections. Studies have shown that patients with higher neutralizing antibody titers at the onset of HPS are more likely to survive or experience milder disease outcomes [5,6,7,8]. Additionally, neutralizing antibodies persist for extended periods after recovery, underscoring their long-term significance in immunity [7,8].

Working with authentic hantaviruses requires biosafety level 3 (BSL-3) containment, presenting significant biosafety and logistical challenges. To address these limitations, surrogate systems that replicate authentic hantavirus infection under BSL-2 conditions have been developed. While lentivirus [9,10] and vesicular stomatitis virus (VSV) pseudotype [11,12,13] systems have provided valuable insights, their limitations in glycoprotein expression and viral particle production highlight the need for improved alternatives [14,15,16]. A recombinant VSV replicating system addresses these challenges by enabling the efficient, high-level expression of hantavirus glycoproteins while maintaining biosafety and biological relevance [17,18]. This system provides a scalable and reliable platform for neutralization assays, allowing for the precise evaluation of antibody responses.

Previously, we developed a recombinant chimeric virus, VSV-SEOV-ZsG, which expresses the glycoprotein of Seoul virus (SEOV) along with the fluorescent protein ZsGreen (ZsG) in infected cells [18]. This chimeric virus was successfully used for antiviral screening and investigating hantavirus entry [18]. In this study, we generated two new VSV chimeric viruses expressing SNV (VSV-SNV-ZsG) and ANDV (VSV-ANDV-ZsG) glycoproteins, respectively. These chimeric viruses were evaluated in neutralization assays using previously characterized monoclonal antibodies [12] and archived clinical serum samples. Additionally, we compared the neutralization titers obtained using the VSV chimeric viruses with those from authentic wild-type hantaviruses in a BSL-3 containment. The comparable results of the two systems support the use of these VSV surrogates as safe, efficient, and biologically relevant alternatives for studying hantavirus neutralization, advancing our ability to evaluate immune responses and therapeutic candidates.

## 2. Materials and Methods

### 2.1. Biosafety

All experiments involving infectious hantavirus material were conducted in BSL-3 facilities following established biosafety protocols. Work with VSV chimeric virus was approved by the CDC Institutional Biosafety Committee to be undertaken at BSL-2.

### 2.2. Cells and Viruses

HuH-7 (from Apath LLC, Brooklyn, NY, USA) and Vero-E6 cells (CRL-1586, ATCC, Manassas, VA, USA) were cultured in Dulbecco’s Modified Eagle’s Medium (DMEM) supplemented with 5% fetal calf serum, penicillin (100 U/mL), and streptomycin (100 μg/mL). HuH-7 cells were further supplemented with 1× non-essential amino acids. ANDV (strain Chile 9717869) and SNV (strain MMR11) were propagated in Vero-E6 cells as described previously. Monoclonal antibodies SNV-53 and ANDV-44 were kindly provided by James Crowe [12].

### 2.3. Patient Serum Sample Selection

CDC archived samples are residual clinical samples that were approved for use in this research by the Centers for Disease Control and Prevention Institutional Review Board (IRB Protocol #7276).

### 2.4. IgG ELISA

SNV-specific IgG titers were measured using ELISA as previously described [6,19]. Briefly, serum samples were serially diluted (4-fold dilutions) from 1:100 to 1:6400. Slurries of SNV-infected Vero-E6 cells served as the test antigen. Adjusted optical density (OD) values were calculated by subtracting the control antigen OD from the SNV-specific OD for each dilution. Samples were considered positive for SNV-specific IgG if the adjusted sum OD value exceeded a defined threshold, and titers were reported as reciprocal values of the highest positive dilution. The selected sera included samples with IgG titers ranging from 50 to 6400, representing a wide range of humoral responses. This selection allowed for further correlation with functional neutralization activity measured in authentic hantavirus neutralization assays.

### 2.5. Generation of VSV Chimeric Viruses

The VSV genome was modified to replace the native VSV glycoprotein (G) with the glycoprotein precursor (GPC) of SNV (NC_005215), ANDV (NC_003467), or SEOV (NC_005237). Each construct also contained a ZsG fluorescent marker for easy detection of infection. VSV chimeric viruses were rescued as described previously [18]. Briefly, VSV chimeric viruses were generated by transfecting HuH-7 cells with pC-VSV-L (expressing VSV polymerase), pC-VSV-N (expressing VSV nucleoprotein), pC-VSV-P (expressing VSV phosphoprotein), and pC-T7 (expressing T7 RNA polymerase), along with one of the following: pVSV-SNV-ZsG (SNV-GPC), pVSV-ANDV-ZsG (ANDV-GPC), or pVSV-SEOV-ZsG (SEOV-GPC). The supernatants were harvested, clarified by centrifugation, and passaged twice in HuH-7 cells to amplify the recombinant virus. Viral stocks were sequenced to confirm the integrity of the genome.

### 2.6. Neutralization Assays 

Neutralization assays were performed using monoclonal antibodies (SNV-53 and ANDV-44) and archived clinical sera. For monoclonal antibodies, 5-fold serial dilutions were prepared and incubated with VSV-SNV-ZsG or VSV-ANDV-ZsG before infecting HuH-7 cells. For patient sera, serial dilutions were similarly prepared and incubated with VSV-SNV-ZsG for 1 h at 37 °C before infection in BSL-2. After 72 h, infection was quantified by measuring ZsGreen fluorescence using BioTek Synergy Plate Reader (Agilent, Santa Clara, CA, USA). Images were also captured using a BioTek Cytation 3 plate reader (Agilent) to confirm quantification. Percent infectivity was calculated relative to virus-only controls (no antibodies added). Normal human plasma served as the negative control, and uninfected cells were included to normalize background fluorescence. For comparison, neutralization assays using authentic hantaviruses were performed under BSL-3 conditions using identical monoclonal antibodies and archived clinical sera. Neutralizing antibody titers (Nab titers) from archived clinical samples were defined as the highest serum dilution resulting in a 50% reduction in infected cells, while the EC₅₀ values for monoclonal antibodies represented the concentration required to achieve the same level of inhibition; both metrics were calculated using a 4-parameter nonlinear regression analysis in GraphPad Prism Version 8.0.2.

For the neutralization assays with authentic SNV and ANDV replication, HuH-7 cells were seeded at 1 × 10^4^/well in 96-well plates one day before infection. Diluted sera or monoclonal antibodies were mixed with authentic SNV or ANDV as described above and added to the cells under BSL-3 conditions. After 72 h, the infected monolayers were fixed with 10% formalin, washed 3 times with phosphate-buffered saline (PBS), and permeabilized with 0.1% (*v*/*v*) Triton X-100 in PBS for 10 min at room temperature. Viral NP was detected with a polyclonal antibody against ANDV NP that cross-reacts with SNV NP. Primary antibodies were detected with goat anti-mouse Alexa 488. Cells were stained with NucBlue (Life Technologies, Carlsbad, CA, USA), and immunofluorescence microscopy was performed using an Operetta high-content imaging system (Revvity, Waltham, MA, USA) [20]. All experiments were conducted in triplicate, and all values were normalized to virus-only controls for analysis.

## 3. Results

### 3.1. Neutralization Assays Using Monoclonal Antibodies and Archived Clinical Sera 

**Figure 1:**
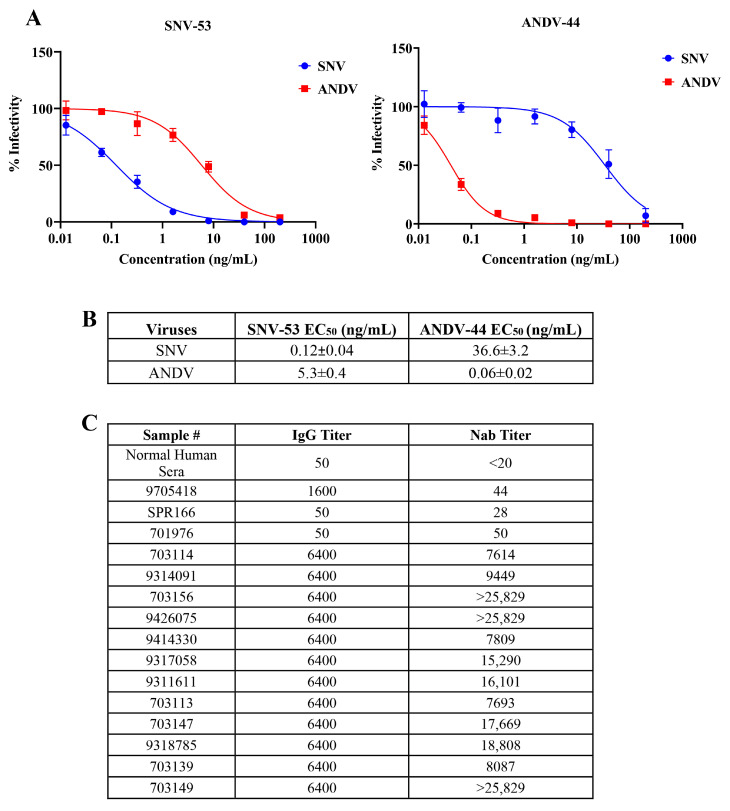
Neutralization efficiency of anti-New World hantavirus monoclonal antibodies and patient serum samples against authentic hantaviruses. (**A**) Dose-dependent inhibition of authentic SNV and ANDV by SNV-53 (**left**) and ANDV-44 (**right**) monoclonal antibodies. (**B**) EC₅₀ of SNV-53 and ANDV-44 against authentic hantaviruses. (**C**) IgG and Nab titers as determined in sera of SNV-infected patients and control samples.

Assays were conducted using authentic SNV and ANDV to evaluate the neutralization titers of monoclonal antibodies SNV-53 and ANDV-44, as well as sera from SNV-infected patients. First, we tested the previously published antibodies (kindly provided by James Crowe, Vanderbilt University) [12] in a neutralization assay using authentic hantaviruses in our BSL-3 laboratory; immunofluorescence staining and microscopy were used to detect the virus. Serially diluted monoclonal antibodies were mixed with authentic SNV or ANDV and added to the cells in BSL-3. After 72 h, the infected monolayers were fixed with 10% formalin, the viral NP was detected with a polyclonal antibody against ANDV (which also recognizes SNV), and immunofluorescence microscopy was performed. The SNV-53 demonstrated strong neutralizing activity against SNV, with an EC_50_ value of 0.12 ± 0.04 ng/mL and limited cross-neutralization against ANDV (EC_50_ = 5.3 ± 0.4 ng/mL) (Figure 1A,B), indicating restricted cross-reactivity between these hantaviruses. Conversely, ANDV-44 potently neutralized ANDV (EC_50_ = 0.06 ± 0.02 ng/mL) while displaying a significantly lower neutralization of SNV (EC_50_ = 36.6 ± 3.2 ng/mL). These findings corroborate prior studies on the specificity of these monoclonal antibodies.

To evaluate neutralizing antibody responses in SNV-infected individuals, archived clinical samples were selected based on IgG titers, determined by IgG ELISA (Figure 1C). While all the samples that exhibited a high titer on the IgG ELISA demonstrated neutralization activity, the Nab titers (defined here as the serum dilution required to achieve 50% neutralization of viral infectivity rather than as ng of antibody, as in the previous section) varied considerably, indicating that IgG titers alone may not fully predict neutralization potency. For example, serum sample 703156 exhibited high neutralization activity, with an Nab titers value exceeding 25,829, highlighting its strong functional antibody response. In contrast, despite having an identical IgG titer of 6400, sample 9314091 showed a much lower Nab titers value of 9449. Similarly, sample 703113 displayed moderate neutralization (Nab titers = 7693), whereas sample 703147 achieved higher potency, with an Nab titers of 17,669. These findings demonstrate considerable variability in the neutralization titers among samples with identical IgG levels and highlight the importance of using Nab titers as a key metric for assessing the functional capacity of neutralizing antibodies in hantavirus-infected individuals.

In contrast, and as expected, the low-IgG-titer serum samples exhibited weaker neutralization activity, as seen with SPR166, which has a Nab titers value of 28 (Figure 1C). Normal human serum served as the negative control and showed no detectable neutralization (<20), confirming the assay’s specificity.

The results highlight the reproducibility of the previously published findings [12] on these monoclonal antibodies and demonstrate the variability in the neutralization potency among patient sera with high IgG titers.

The rescue and characterization of recombinant VSV chimeric viruses expressing hantavirus glycoproteins were then conducted.

**Figure 2:**
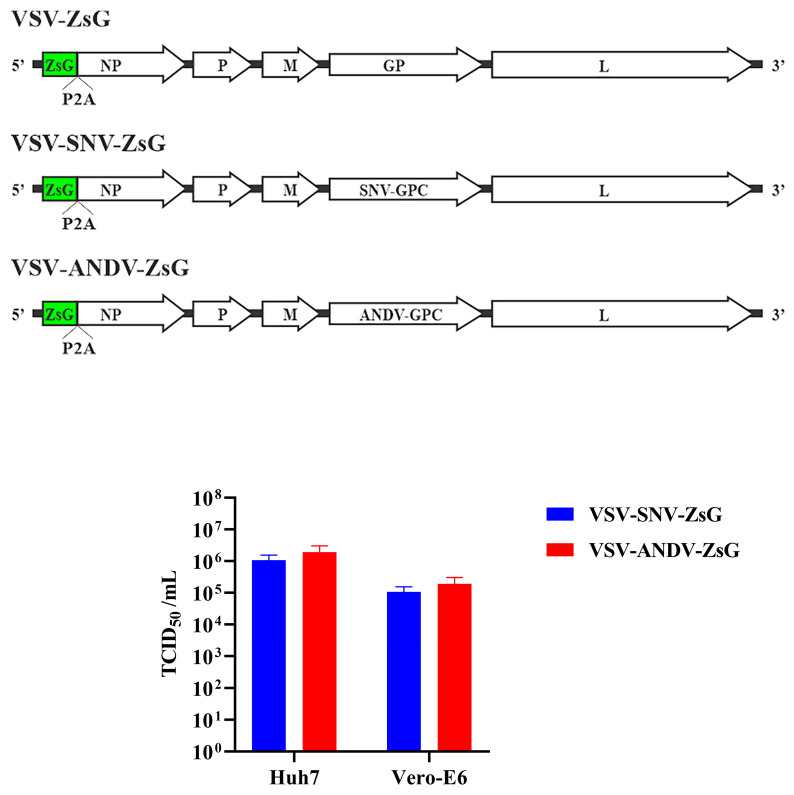
Recombinant VSV chimeric viruses expressing SNV and ANDV glycoproteins. **Top panel**: schematic of VSV genome modified to replace VSV-GP with SNV-GPC or ANDV-GPC. **Bottom panel**: TCID_50_/mL values for VSV-SNV-ZsG and VSV-ANDV-ZsG in Vero-E6 and Huh-7 cells.

The schematic in the top panel of Figure 2 shows the design of the VSV chimeric viruses. In these recombinant viruses, the native VSV glycoprotein is replaced with SNV-GPC or ANDV-GPC. The viruses also incorporate the fluorescent ZsG marker, which facilitates the detection of the virus in neutralization assays under BSL-2 and without the need for fixing and staining monolayers with specific antibodies. Moreover, ZsG allows for the real-time tracking of viral replication at multiple intervals using instruments like the Synergy plate reader without disrupting the cells.

The functionality of the chimeric viruses was tested in Vero-E6 and Huh-7 cell lines to evaluate their suitability for neutralization studies. The chimeric viruses enable viral entry mediated by the hantavirus GPC, mimicking an authentic hantavirus infection, and were tested for infectivity to confirm the successful rescue of the VSV system. First, we titered the rescued virus on monolayers of Vero-E6 or Huh-7 cells. Since we observed higher titers on the Huh-7 cells, these were used in the subsequent neutralization assays.

### 3.2. Testing the Recombinant VSV-Based Hantavirus Surrogate System Using Neutralization Assays

**Figure 3:**
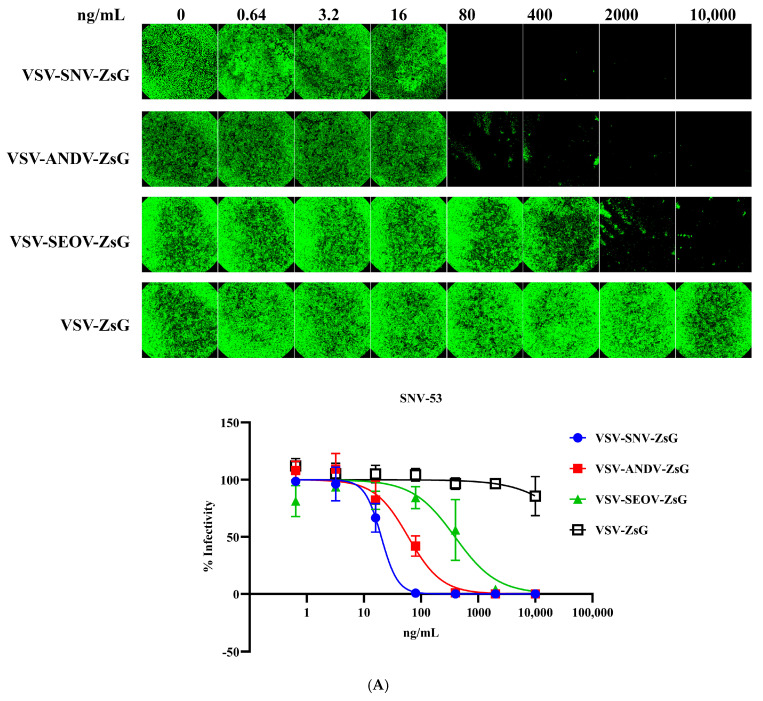

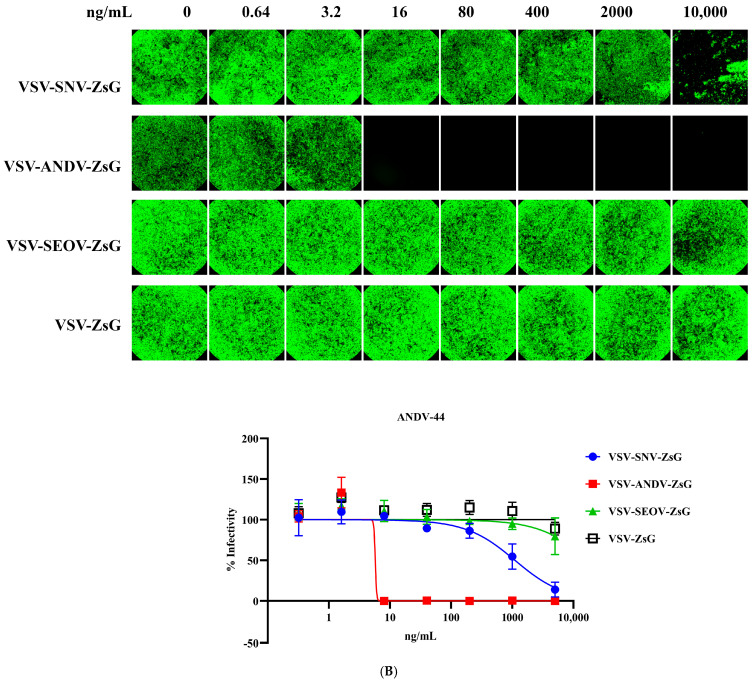
Neutralization of VSV-SNV-ZsG and VSV-ANDV-ZsG by monoclonal antibodies. Dose-dependent reduction in (**A**) VSV-SNV-ZsG and (**B**) VSV-ANDV-ZsG infectivity by SNV-53 and ANDV-44.

Building on the previous results, the recombinant VSV system expressing hantavirus glycoproteins (SNV-GPC or ANDV-GPC) was further evaluated for its utility in neutralization assays using the above-tested monoclonal antibodies. The neutralization assays using VSV-SNV-ZsG and VSV-ANDV-ZsG are shown in Figure 3A,B and Table 1. Figure 3A,B show representative fluorescence microscopy images of infected cells treated with varying concentrations of monoclonal antibodies. The bottom panels summarize these results, highlighting the differences in the neutralization efficiency. The EC_50_ values derived from these curves are summarized in Table 1.

### 3.3. Neutralization of VSV-SNV-ZsG Using Monoclonal Antibodies

Figure 3A shows the neutralization of VSV-SNV-ZsG by monoclonal antibody SNV-53. The infectivity curve demonstrates a clear dose-dependent reduction in infectivity with increasing concentrations of SNV-53. The EC_50_ of SNV-53 against VSV-SNV-ZsG was 20.09 ng/mL, indicating strong neutralization efficiency. As expected, ANDV-44 exhibited limited neutralization against heterologous SNV virus, with a much higher EC_50_ value (59.47 ng/mL), highlighting its reduced specificity for SNV glycoproteins. ANDV-44 strongly neutralized VSV-ANDV-ZsG, with an EC_50_ value of 5.732 ng/mL, but, as expected, SNV-53 showed significantly weaker neutralization against this virus (EC_50_ = 18,206 ng/mL), indicating its reduced specificity for ANDV glycoproteins (Figure 3B). These results align with the specificity observed in the authentic hantavirus assays, validating the use of the VSV system for neutralization studies.

The specificities of the tested monoclonal antibodies were demonstrated by low neutralization titers against heterologous viruses. Neither SNV-53 nor ANDV-44 effectively neutralized VSV-SEOV-ZsG, as evidenced by high EC_50_ values (391.1 ng/mL for SNV-53 and >10,000 ng/mL for ANDV-44). While the neutralization of SEOV was less potent than that of SNV, it is notable that there was still an impressive cross-neutralizing activity, consistent with previously published reports [12,15].

Table 1 summarizes the EC_50_ values for the neutralization of VSV-based chimeric viruses by SNV-53 and ANDV-44. While the EC_50_ values for the recombinant VSV system differ slightly from those observed with authentic hantaviruses, the data demonstrate the robust neutralization efficiency of SNV-53 and ANDV-44 against their respective targets. These findings indicate that the VSV-SNV-ZsG and VSV-ANDV-ZsG chimeric viruses are suitable surrogates for neutralization assays under BSL-2 conditions. VSV-SEOV-ZsG was not neutralized, further supporting the specificity of the monoclonal antibodies. These results validate the use of the VSV-based system for neutralization assays in BSL-2 settings. Despite differences in the EC_50_ values compared to authentic hantavirus assays, the recombinant chimeric viruses are potently neutralized by these antibodies, confirming their utility as safe and accessible alternatives for evaluating neutralizing antibodies.

### 3.4. Neutralization Potency of Archived Clinical Samples Using VSV-SNV-ZsG 

**Figure 4:**
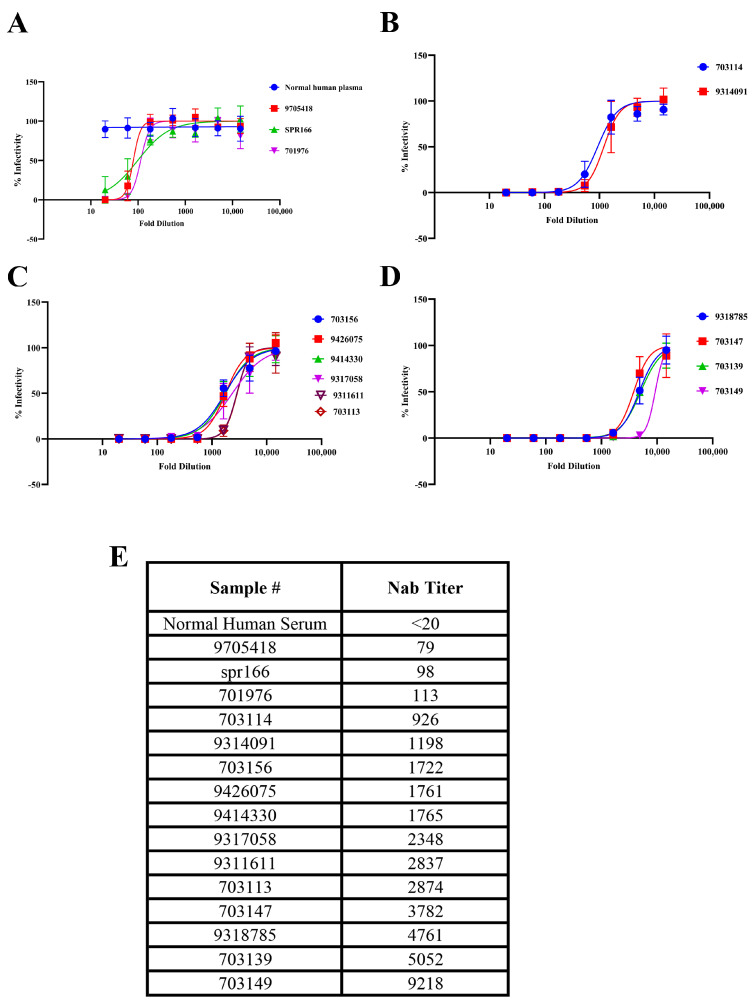
Neutralization potency of clinical samples against VSV-SNV-ZsG. Neutralization curves with sera grouped by potency from weakest (**A**–**D**) to strongest. (**E**) Nab titers of all clinical samples.

To evaluate the neutralization potency of patient sera using the recombinant VSV-SNV-ZsG system, the serum sample data were divided into four panels. As shown in Figure 4, the samples were organized according to the neutralization titers, ranging from weakest (Figure 4A) to strongest (Figure 4D), based on their ability to neutralize VSV-SNV-ZsG at various serum dilutions. The EC_50_ values, representing the dilutions needed to neutralize 50% of viral infectivity, were used as the primary metric for these categorizations. Figure 4E summarizes the Nab titers of all the clinical samples.

The top-left panel includes the samples with the lowest EC_50_, such as SPR166 and 9705418. These samples required lower dilutions of sera to achieve 50% neutralization, indicating low neutralization titers. This suggests that the levels of neutralizing antibodies in these samples are insufficient to effectively inhibit viral infectivity at lower serum concentrations. This categorization aligns with the expectation for them to be weak neutralizers, as observed in the corresponding BSL-3 assays with authentic SNV.

### 3.5. Comparison of Neutralization Potency in Archived Clinical Samples Using VSV-SNV-ZsG and Authentic SNV Assays

To further evaluate the relevance of the VSV-based pseudovirus system described in this study, we compared the Nab titers obtained using VSV-SNV-ZsG with those derived from assays employing authentic SNV. As presented in Table 2, the archived human serum samples were categorized into three groups based on their Nab titers: high (>1000), moderate (100–1000), and low (<100). A consistent neutralization response was observed in the majority of the samples (15 out of 17) across both assay systems. The two exceptions—samples 701976 and 703114—exhibited moderate Nab titers against VSV-SNV-ZsG, but showed low or high titers, respectively, when tested against authentic SNV.

## 4. Discussion

Previous studies have shown that, although IgG titers are generally elevated during the course of HPS, they do not perfectly correlate with disease severity. In contrast, neutralizing antibody titers strongly correlate with patient survival [5,7], making them a more reliable predictor of disease outcome than IgG levels alone. Our assay provides a more direct measure of protective immunity and may serve as a valuable tool for disease monitoring in BSL-2 settings.

Unlike the pseudotyped VSV systems used in previous studies [12,14], our recombinant virus express ANDV or SNV glycoproteins along with the ZsGreen fluorescent reporter, allowing for the real-time monitoring of neutralization assays without requiring cell harvest and lysis. This further enhances the high-throughput quantification, making it suitable for large-scale studies of neutralizing antibodies or antiviral compounds.

This study presents the development and application of recombinant VSV-based systems expressing hantavirus glycoproteins as a safe and effective alternative for neutralization assays. The VSV chimeric viruses expressing SNV and ANDV glycoproteins were evaluated using previously characterized monoclonal antibodies and patient sera. These VSV-based constructs were robustly neutralized by SNV-specific (SNV-53) and ANDV-specific (ANDV-44) monoclonal antibodies. The EC50 values obtained from these assays closely correlated with those from authentic hantavirus systems, further validating the utility of VSV chimeric viruses for hantavirus neutralization studies. The results were strongly consistent with neutralization assays conducted using authentic hantaviruses in BSL-3 conditions, validating the system as a reliable surrogate for such assays. Moreover, the archived serum samples from SNV-infected individuals exhibited comparable neutralization profiles when tested with both the VSV chimeric viruses and authentic hantavirus assays, supporting the VSV surrogate system as a reliable alternative to wild-type viruses for neutralization studies. Consistent with previous reports [5,7,8], all the archived samples that exhibited high IgG titers (measured by ELISA) also demonstrated moderate-to-high neutralization titers against both authentic hantaviruses and the VSV surrogates (Figure 1C and Figure 4B, and Table 2). However, as observed in Figure 1C and Figure 4B, most of the samples neutralized the authentic viruses at a higher dilution than required for the VSV surrogates. We hypothesize that a higher antibody concentration was necessary to neutralize the VSV surrogates due to the faster replication of VSV compared to authentic hantaviruses. Alternatively, authentic hantaviruses may present their glycoprotein in a different conformation, allowing for more efficient antibody binding of antibodies and neutralization.

The extensive safety record of VSV-based vaccine platforms, including the VSV–Ebola vaccine [21], supports the use of a replication-competent VSV expressing hantavirus glycoproteins in BSL-2 settings. Multiple studies have demonstrated the biosafety of these systems in animal models [22,23,24,25,26], reinforcing their suitability for controlled laboratory use.

While authentic hantavirus systems remain the gold standard for neutralization studies, VSV systems provide a safe, scalable, and accessible platform for high-throughput screening of neutralizing antibodies, particularly in laboratories without BSL-3 containment facilities. This study highlights the importance of combining both approaches to achieve a comprehensive understanding of neutralizing antibody responses. A recombinant system provides a critical platform for advancing serological and antiviral research, supporting the development of therapeutic antibodies and vaccines for hantavirus infections.

## Figures and Tables

**Table 1:** EC_50_ of NWH-reactive monoclonal antibodies against VSV chimeric hantaviruses.

	SNV-53 EC_50_ (ng/mL)	ANDV-44 EC_50_ (ng/mL)
VSV-ZsG	>10,000	>10,000
VSV-SNV-ZsG	20.09	1104
VSV-ANDV-ZsG	59.47	5.7
VSV-SEOV-ZsG	391.1	>10,000

**Table 2:** Comparison of neutralization potency in archived clinical samples using VSV-SNV-ZsG and authentic SNV assays.

Clinical Sample #	Neutralization Potency SNV/VSV-SNV-ZsG
Normal Human Sera	Low/Low
9705418	Low/Low
SPR166	Low/Low
701976	Low/Moderate
703114	High/Moderate
9314091	High/High
703156	High/High
9426075	High/High
9414330	High/High
9317058	High/High
9311611	High/High
703113	High/High
703147	High/High
9318785	High/High
703139	High/High
703149	High/High

## Data Availability

The original contributions presented in this study are included in the article. Further inquiries can be directed to the corresponding authors.
